# The Relationship Between Spiritual Well-Being and Fear of COVID-19 Among Cancer Patients in Turkey

**DOI:** 10.1007/s10943-025-02303-9

**Published:** 2025-04-10

**Authors:** Nurgül Karakurt, Hatice Cecen Celik, Yasemin Erden

**Affiliations:** 1 Nursing Department, Faculty of Health Sciences, Erzurum Technical University, Erzurum, Turkey; 2 Emergency and Disaster Management Department, Faculty of Health Sciences, Erzurum Technical University, Erzurum, Turkey

**Keywords:** Cancer patients, Faith, Fear of COVID-19, Spiritual well-being

## Abstract

This study focuses on the relationship between the faith sub-dimension of spiritual well-being and COVID-19-related fear among cancer patients. The research employs a descriptive, correlational, and cross-sectional approach to understand how faith influences psychological resilience and reduces fear in this population during the pandemic. The study was conducted with 261 cancer patients treated at a Health Application and Research Center in Turkey from March to May 2022. Data were collected using the Faith sub-dimension and the coronavirus fear scale (C19P-S). The findings reveal a significant negative relationship between Faith sub-dimension and COVID-19 fear, suggesting that Faith plays a pivotal role in enhancing psychological well-being. In line with these findings, the study recommends integrating spiritual support into the psychological care processes for cancer patients, focusing mainly on faith-based support. Recommended practical applications include spiritual counseling services provided through telemedical systems and the establishment of hospital-based spiritual care teams structured to suit cancer patients’ emotional and spiritual needs.

## Introduction

Cancer patients, already struggling with the challenges of a life-changing diagnosis, are facing increased psychological stress during the COVID-19 pandemic. This complex crisis highlights the importance of understanding how spiritual well-being can protect against pandemic-related fear and anxiety. As a highly contagious disease, COVID-19 swiftly escalated into a global public health emergency, exerting effects far beyond physical health and including substantial psychological repercussions (Cao et al., 2020; Yıldırım & Solmaz, 2022). These effects are particularly severe for individuals with chronic conditions such as cardiovascular disease, cancer, and diabetes, who have reported increased levels of fear, depression, and anger (Ing et al., 2020; Wang et al., 2020). This psychological toll has been conceptualized in new terms such as Corona phobia (Arora et al., 2020) and COVID-19 stress Syndrome (Asmundson & Taylor, 2020). For cancer patients, in particular, the heightened anxiety is justified as they face a dual risk: an increased likelihood of contracting COVID-19 and more severe outcomes due to immunosuppression caused by cancer progression or treatments like chemotherapy (Abdihamid et al., 2020). Studies have highlighted that cancer patients during the pandemic were frequently diagnosed with depression, anxiety, and post-traumatic stress disorder (Wang et al., 2020).

In response to these psychological challenges, many individuals living with chronic illnesses turn to spirituality as a coping mechanism. According to Pargament’s religious coping theory, stronger religious-oriented individuals use spiritual coping strategies more effectively. Spirituality is an important element in individuals’ lives and contributes to their understanding, maintenance, and transformation of life experiences. It serves five primary functions: discovering meaning, gaining control, finding comfort through connection with the Creator, fostering closeness with others, and facilitating personal transformation (Pargament et al., 2004). For those facing existential crises, such as life-threatening illnesses, spirituality often becomes central to finding meaning, hope, and resources for resilience (Chatrung et al., 2015).

Interest in exploring the impact of spirituality on physical and mental health is increasing, with evidence suggesting that higher self-reported spirituality is linked to positive physical and psychological outcomes (Delgado-Guay et al., 2021; Jim et al., 2015). Spirituality can be significant throughout life but often becomes especially relevant when individuals face life-threatening illnesses like cancer (Balducci, 2019). Research suggests that spirituality fosters supportive thoughts and emotions, such as safety and comfort, during challenging circumstances (Renz et al., 2018). It acts as an effective positive coping strategy in adjusting to cancer and its symptoms (Cheng et al., 2019). In addition, spirituality can support the adoption of self-management strategies in cancer patients (Yeager et al., 2016). For example, a study conducted by Delgado-Guay et al. (2021) revealed that spirituality and religiosity were positively associated with the management and coping process of physical and emotional symptoms in advanced cancer patients in Latin America. This finding reinforces the idea that spiritual support may benefit cancer patients.

The spiritual needs of individuals significantly increased during the COVID-19 pandemic, with the role of spirituality becoming more prominent (González-Sanguino et al., 2020). Religious coping, in particular, is shown to shield individuals from the adverse effects of stress, while spiritual coping strategies have proven effective in reducing stress, regardless of its intensity (Pargament et al., 2004; Xu, 2016). Moreover, spirituality has been identified as a crucial resource for individuals with chronic illnesses during the pandemic, positively influencing their treatment outcomes (Wu & McGoogan, 2020). Research has consistently shown that spirituality provides significant benefits to individuals dealing with chronic illnesses. These include improved mental health, enhanced resilience, and better management of physical and emotional challenges (Hosseini et al., 2013; Torabi et al., 2017). Among cancer patients specifically, engaging with spirituality has been shown to positively impact their mental health and overall well-being (Albayrak et al., 2019; Boscaglia et al., 2005; Choumanova et al., 2006). During the COVID-19 pandemic, spirituality has been increasingly recognized as a vital resource for resilience. Many individuals have relied on spiritual well-being to cope with the psychological stresses brought on by the pandemic (González-Sanguino et al., 2020; Pargament et al., 2004; Xu, 2016). Spiritual practices have been found to reduce stress and promote recovery, particularly among hospitalized patients. For instance, patients who engaged with their spirituality experienced shorter hospital stays and improved overall physical and psychological quality of life (Wu & McGoogan, 2020).

Despite its importance, research has revealed a significant gap in access to spiritual care for individuals with chronic diseases during the COVID-19 pandemic. The reasons for this gap include time constraints, inadequate professional training, lack of awareness, cultural differences, and institutional barriers, all of which make it challenging to provide effective spiritual care. In some developed nations, spiritual care is integrated into healthcare services and indicates service quality, yet this integration must be consistent globally. For cancer patients, spirituality has played a pivotal role in fostering hope, finding meaning, and managing existential fears, particularly in the face of pandemic-related anxiety. As COVID-19 continues to evolve with the emergence of new variants, it is increasingly essential to understand the relationship between spiritual well-being and pandemic-related psychological distress. This understanding is critical not only to addressing the mental health needs of cancer patients but also to guiding the development of interventions and tools designed to mitigate their psychological distress and improve overall well-being. Therefore, this study seeks to contribute to this understanding by examining the interplay between spiritual well-being and COVID-19-related fear in cancer patients. Specifically, it aims to provide insights that can guide the development of interventions and tools to mitigate psychological distress in this population.

### Research Questions

- What are the levels of the Faith sub-dimension of the FACIT-Sp among cancer patients during the COVID-19 pandemic?
- What are the levels of COVID-19-related fear among cancer patients?
- Is there a significant relationship between the Faith sub-dimension of FACIT-Sp and COVID-19-related fear among cancer patients?
- How do demographic and clinical factors influence the relationship between the Faith sub-dimension of the FACIT-Sp and COVID-19-related fear in cancer patients?

By addressing these questions, this study aims to bridge gaps in knowledge and provide practical guidance for integrating spiritual care into comprehensive cancer treatment.

## Materials and Methods

### Type of Research

This descriptive and correlational study explores the association between the Faith sub-dimension of FACIT-Sp and fear of COVID-19 among cancer patients.

### Place and Time of Research

This study was conducted with cancer patients receiving treatment in the outpatient chemotherapy unit of a university hospital in eastern Turkey between March and May 2022.

### Population and Sample of the Research

The research employed the simple random sampling method, a fundamental and commonly utilized technique in sampling. In this approach, every individual in the population has an equal chance of being selected, and the selection process is entirely random. Simple random sampling effectively ensures that the sample accurately represents the population, and its reliability in reflecting the population can be assessed with confidence (Noor et al., 2022). For this study, a list of all patients registered at the hospital who met the research criteria was first compiled. This list included every individual who qualified for the study. Following this, random selections were made using a computer-generated random number list. The individuals selected for participation were chosen based on the random numbers produced by the software.

To determine the sample size, the G POWER 3.1.9.7 package program was used to calculate the adequate sample size. Since no similar study was found in the literature, an a priori power analysis was performed, assuming a medium effect size of 0.3. Based on an alpha of 0.05 and a power of 0.95, the power analysis determined that the sample size should be 260 (Durmus & Durar, 2022). This study was completed with 261 patients.

#### Inclusion Criteria for the Study

- Being 18 years of age or older
- Having been diagnosed with cancer for at least 6 months
- Being able to understand and speak Turkish.

#### Exclusion Criteria for the Study

- Having a diagnosis of a psychiatric disorder that may interfere with study participation.
- Experiencing significant cognitive or communication impairments that hinder the ability to provide informed consent or complete the study questionnaires.
- Having a co-existing severe medical condition unrelated to cancer could affect participation in the study.

### Data Collection Tools

#### Personal information form

The personal information form prepared by researchers consists of 9 questions (age, gender, marital status, employment status, duration of illness, time since the last treatment, COVID-19 status, and hospitalization due to COVID-19).

#### Coronavirus Fear Scale (C19P-S)

The C19P-S was produced by Ahorsu et al. (2022) to measure the fear that individuals may hold as to COVID-19, with a Turkish version of the C19P-S having been developed (and ascertained as to its reliability and validity) by Ladikli et al. (2020). The scale consists of seven items with a single factor and a five-point Likert type (1 = Strongly disagree; 5 = Strongly agree). The lowest score that can be obtained from the scale is 7, while the highest score is 35, and a high score obtained from the scale indicates a high fear of COVID-19. Ladikliet al. (2020), in considering the Cronbach’s Alpha coefficient of the C19P-S, found this to be 0.86. In the present research, this has been identified at 0.94.

#### Functional Assessment of Chronic Illness Therapy-Spiritual Well-Being Scale (FACIT-Sp)

The FACIT-Sp was produced by Peterman et al. (2002) to illustrate the level of spiritual well-being held by individuals experiencing chronic health problems or living with cancer. A Turkish version of the FACIT-Sp was developed (and ascertained as to its reliability and validity) by Aktürk et al. (2017). The FACIT-Sp comprises 12 items and employs a five-point Likert scale spanning 0 = Not At All, 1 = = A Little Bit, 2 = Somewhat, 3 = Quite a Bit, and 4 = Very Much. The FACIT-Sp has three aspects, divided between Meaning (Items 2, 3, 5, 8), Peace (Items 1, 4, 6, 7) and Faith (Items 9, 10, 11, 12). Faith Sub-dimension of FACIT-Sp: The Faith sub-dimension (items 9–12) assesses trust in a higher power and religious Faiths. Higher scores indicate greater Faith.

The FACIT-Sp Scale used in the study consists of three sub-dimensions: Meaning, Peace, and Faith. Analyses were conducted at the sub-dimension level, considering the risk of contamination emphasized by Koenig and Carey (Koenig & Carey, 2024). Particular attention was paid to the meaning sub-dimension because it may overlap with psychological structures.

### Data Collection

Data were collected through face-to-face interviews conducted by trained researchers to minimize bias and ensure consistency in question presentation. The interviews were scheduled at times that did not conflict with patients’ meals, sleep, or visiting hours, and each session lasted approximately 5–10 min. Verbal and written consent was obtained from each participant before data collection, ensuring ethical compliance.

### Data Evaluation

Data analysis was performed using SPSS 22 software. The normality of distributions was assessed through skewness and kurtosis values, along with histogram charts. The reliability of the scales was evaluated using Cronbach’s Alpha coefficient. Descriptive statistics were summarized using frequency and percentage analyses. The Independent Samples t-test and Mann–Whitney U Test were utilized to compare groups. At the same time, One-Way ANOVA and Kruskal–Wallis tests were employed for comparisons across multiple groups. Correlation analysis assessed relationships among variables, with a significance level set at 0.05.

### Ethical Considerations

The research gained ethical approval from the Erzurum Technical University Scientific Research and Publication Ethics Committee (28.02.2022), and all research participants were required to provide written informed consent as to their involvement in the research following the conveyance by the researcher of comprehensive information regarding the purpose and design of the study. The study was designed so that the research participants would experience no harm.

## Results

When the distribution of descriptive characteristics of individuals included in the study was investigated, the mean age of the participating patients was 57.11 ± 11.67, and 57.5% were women. Also, 90% of the patients were unemployed, 92.7% were married, 36% were diagnosed with cancer between 6 and 11 months, 74.4% had spent less than 3 months after their last treatment, and 39.1% were in the second stage of the disease. Moreover, it was determined that 63.2% of the patients did not have COVID-19, and 14.6% of the patients who had COVID-19 were hospitalized (Table 1).

**Table 1:** Comparison of patients’ total mean scores on the fear of COVID-19 scale and faith sub-dimension according to descriptive characteristics

		Coronafobia scale	Faith sub-dimension
		X ± S.d	X ± S.d
Gender	Female	18.21 ± 7.76	12,48 ± 2.48
	Male	17.85 ± 7.361	12.13 ± 2.48
	TEST *p*	t = .385 *p* = .700	t = 1.140 *p* = .255
Age	26–50^1^	18.40 ± 5.40	10.98 ± 2.29
	50–70^2^	17.33 ± 8.34	12.70 ± 2.46
	70 ≥ ^3^	20.00 ± 7.24	12.96 ± 2.16
	TEST *p*	F = 2.296 *p* = .103	**F = 13.612** ***p* = .001**
	Post hoc	–	**2–3 > 1**
Employment status	Employed	15.65 ± 4.70	11.54 ± 1.10
	Unemployed	18.32 ± 7.80	12.42 ± 2.58
	TEST *p*	U = 2452.50 *p* = .097	U = 2610.00 *p* = .212
Marital status	Married	18.45 ± 7.53	12.49 ± 2.20
	Single	13.00 ± 6.45	10.26 ± 4.40
	TEST *p*	**U = 1384.00** ***p* = .004**	U = 1714.50 *p* = .059
Time since cancer diagnosis	Less than 5 months^1^	15.82 ± 6.10	12.53 ± 2.59
	6–11 months^2^	21.98 ± 6.25	11.83 ± 2.54
	1–2 year^3^	19.40 ± 6.02	11.21 ± 1.20
	2 years and more^4^	13.68 ± 8.31	13.54 ± 2.40
	TEST *p*	**F = 22.49** ***p* = .000**	**F = 10.742** ***p* = .000**
	Post Hoc	**2 > 1–4**	**4 > 2–3**
Time since last cancer treatment	Less than 3 months^1^	18.46 ± 7.81	12.45 ± 2.60
	3–6 months^2^	13.59 ± 6.08	11.06 ± 1.25
	6–12 months^3^	17.06 ± 2.54	12.00 ± .63
	TEST *p*	**KW = 7.298** ***p* = .026**	**KW = 6.262** ***p* = .044**
	Post Hoc	**1 > 2**	**1 > 2**
Stage of disease	Stage 1	15.32 ± 7.10	13.73 ± 1.72
	Stage 2	20.62 ± 6.43	12.01 ± 1.32
	Stage 3	20.78 ± 7.16	11.80 ± 2.69
	Stage 4	12.81 ± 6.63	12.83 ± 3.37
	TEST *p*	**KW = 53.92** ***p* = .000**	**KW = 31.184** ***p* = .000**
	Post Hoc	**2–3 > 1–4**	**1 > 2–3**
Have you had COVID-19?	Yes	19.48 ± 5.53	11.38 ± 2.18
	No	17.23 ± 8.46	12.88 ± 2.48
	TEST *p*	**t = 2.592** ***p* = .010**	**t = -4.952** ***p* = .000**
Have you been hospitalized due to COVID-19?	Yes	20.21 ± 4.86	10.42 ± 2.83
	No	17.69 ± 7.90	12.65 ± 2.27
	TEST *p*	**t = 2.653** ***p* = .010**	**t = -5.402** ***p* = .000**

Statistically significant values are shown in bold (*p* 0.05); *p* 0.001 indicates strong statistical significance

*p* < 0.001 indicates strong statistical significance

^1^ Group 1, ^2^ Group 2, ^3^ Group 3, ^4^ Group 4 — used for post-hoc comparison references

### Clinical Characteristics

The analysis revealed no significant differences between males and females regarding the Faith sub-dimension and the Fear of COVID-19 Scale scores. However, significant differences were found in the Faith sub-dimension scores based on age, while the differences in the Fear of COVID-19 Scale scores were not statistically significant. To determine the source of the difference, a Scheffe Post Hoc test was conducted, indicating that individuals aged 50–70 and those over 70 had significantly higher Faith sub-dimension scores than those aged 26–50 (*p* < .050).

The differences in the scores of the Faith sub-dimension and The Fear of COVID-19 Scale according to employment status were compared using the Mann–Whitney U test and found to be non-significant. (*p* > .050).

The differences in the Fear of COVID-19 Scale scores according to marital status were compared using the Mann–Whitney-U test and were found to be significant in favor of the unmarried group. However, the differences in the Faith sub-dimension scores were found to be non-significant. (*p* > .050).

Differences in the Faith sub-dimension and The Fear of COVID-19 Scale scores based on the duration since the cancer diagnosis were examined using ANOVA analysis and found to be statistically significant. A Scheffe Post Hoc test was conducted to identify the specific groups contributing to these differences. Results indicated that individuals diagnosed with cancer 6–11 months ago had significantly higher scores on The Fear of COVID-19 Scale compared to those diagnosed less than 5 months ago (*p* < .050). Furthermore, those diagnosed with cancer 2 years or more ago had significantly higher scores on the Faith sub-dimension compared to individuals diagnosed 6–11 months and 1–2 years ago (*p* < .050).

Differences in the Faith sub-dimension and The Fear of COVID-19 Scale scores based on the duration since the last cancer treatment were analyzed using the Kruskal–Wallis test, and statistically significant differences were found. To determine which groups contributed to these differences, a Dunn-Bonferroni Post Hoc test was performed. The findings revealed that individuals whose last cancer treatment was less than 3 months ago scored significantly higher on the Faith sub-dimension and The Fear of COVID-19 Scale than those whose treatment was 3–6 months ago (*p* < .050).

Differences in Faith sub-dimension and The Fear of COVID-19 Scale scores based on the stage of the disease were examined using the Kruskal–Wallis test, and the results were statistically significant. A Dunn-Bonferroni Post Hoc test identified that individuals in stages 2 and 3 had significantly higher scores on The Fear of COVID-19 Scale than in stages 1 and 4 (*p* < .050). Conversely, individuals in stage 1 had significantly higher scores on the Faith sub-dimension than those in stages 2 and 3 (*p* < .050).

Differences in the Faith sub-dimension and The Fear of COVID-19 Scale scores based on COVID-19 infection status were analyzed using the t-test, and the results were statistically significant. The data indicated that individuals who had contracted COVID-19 had significantly higher scores on The Fear of COVID-19 Scale and significantly lower scores on the Faith sub-dimension (*p* < .050).

Differences in the Faith sub-dimension and The Fear of COVID-19 Scale scores based on hospitalization due to COVID-19 were also analyzed using the t-test, and statistically significant differences were identified. Individuals hospitalized due to COVID-19 had significantly higher scores on The Fear of COVID-19 Scale and significantly lower scores on the Faith sub-dimension (*p* < .050).

FACIT-Sp Scale and COVID-19 Fear and Sub-Dimensions of FACIT-Sp Scale.

The sub-dimensions of the FACIT-Sp Scale have score ranges between 0 and 12. When examining the mean scores, it was found that meaning scored 9.20 ± 1.84, Faith sub-dimension scored 12.33 ± 2.48, and peace scored 8.95 ± 1.77. It can be stated that the highest mean score was in the Faith sub-dimension, while the lowest was in the Peace sub-dimension, and the mean scores for all sub-dimensions and the total scale were high. The total score range of the Fear of COVID-19 Scale is between 7 and 35. The mean score was 18.06 ± 7.58, indicating the patient's fear of COVID-19 was slightly above average (Table 2).

**Table 2:** Total scores of the fear of COVID-19 scale, and its FACIT-sp scale sub-dimensions (*n* = 261)

FACIT-sp scale sub-dimensions	Min–Max	Mean ± SD
Meaning	2–12	9.20 ± 1.84
Peace	4–12	8.95 ± 1.77
Faith	3–16	12.33 ± 2.48
The fear of COVID-19	7–35	18.06 ± 7.58

The score ranges that can be obtained from the sub-dimensions of the FACIT-Sp Scale are between 0 and 12, and when the score averages are examined, it is seen that meaning is 9.20 ± 1.84, Faith sub-dimension is 12.33 ± 2.48, and peace is 8.95 ± 1.77. It can be said that the highest mean score is for the faith sub-dimension, the lowest mean is for peace, and the average scores from all dimensions and the total of the scale are high. The total score ranges that can be obtained from the Fear of COVID-19 Scale are between 7 and 35, and when the score average is examined, it can be said that it is 18.06 ± 7.58, and the patients' fear of COVID-19 is slightly above average (Table 2).

### Correlation Analysis

**Fig. 1:**
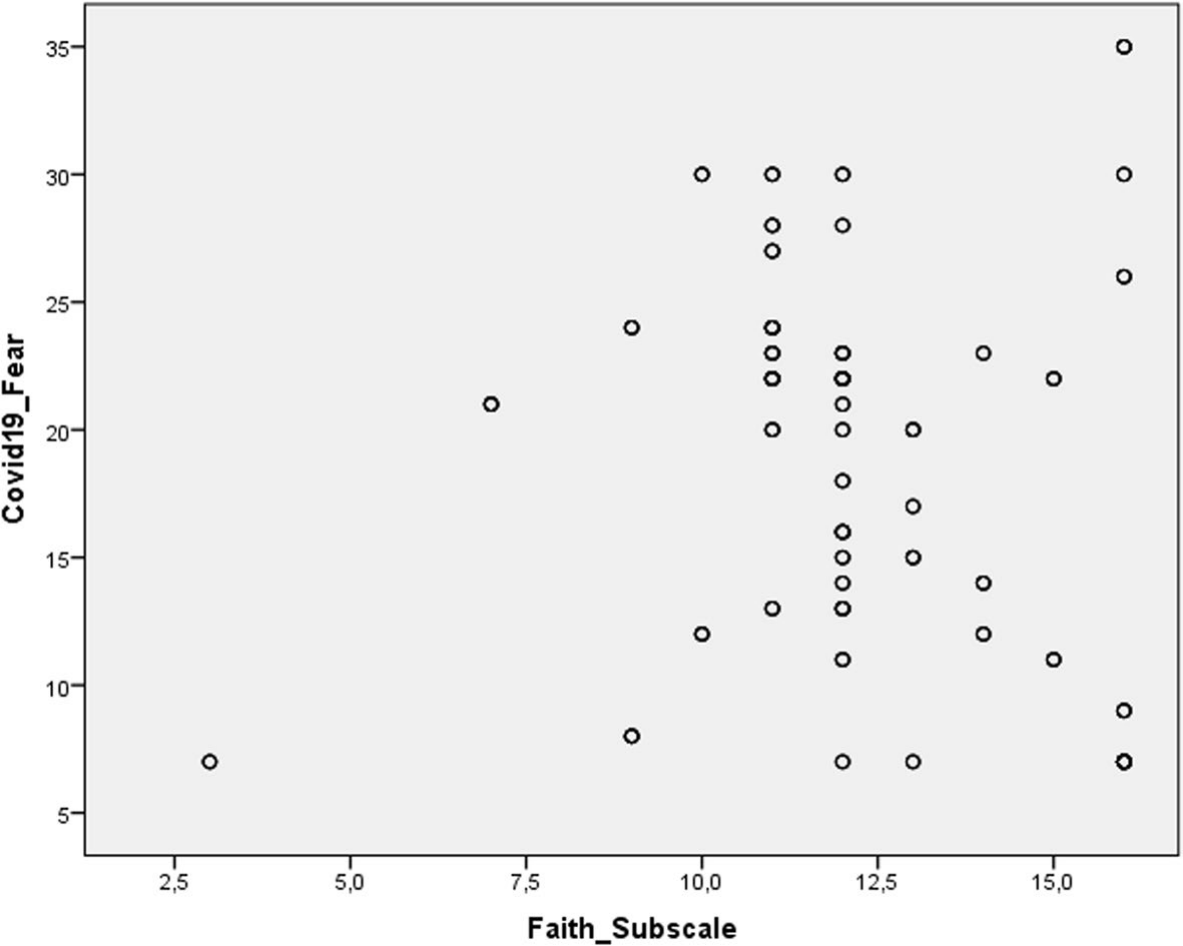
Scatter plot graph of the relationship between COVID-19 fear and faith sub-dimension

The statistical analysis of the relationship between the Faith sub-dimension of the FACIT-Sp Scale and cancer patients’ fear of COVID-19 revealed a significant negative correlation (r = − 0.214, *p* < 0.001). The Faith sub-dimension showed a strong positive correlation with the overall FACIT-Sp score (r = 0.888, *p* < 0.001). The Faith sub-dimension also exhibited significant positive correlations with the meaning (r = 0.667, *p* < 0.001) and peace (r = 0.558, *p* < 0.001) sub-dimensions (Table 3). A scatter plot illustrates the relationship between fear of COVID-19 and Faith sub-dimension (Fig. 1).

**Table 3:** Correlation analysis results for patients’ FACIT-sp sub-dimensions and fear of COVID-19 scale scores

		Meaning sub-dimension	Peace sub-dimension	Faith sub-dimension	Fear of COVID-19 scale
Meaning sub-dimension	r	1	.657^**^	.667^**^	.881^**^
	*p*		**.000**	**.000**	**.000**
Peace sub-dimension	r	.657^**^	1	.558^**^	− .210^**^
	*p*	**.000**		**.000**	**.001**
Faith sub-dimension	r	.667^**^	.558^**^	1	− .214^**^
	*p*	**.000**	**.000**		**.000**
Fear of COVID-19 scale	r	− .192^**^	− .210^**^	− .214^**^	1
	*p*	**.002**	**.001**	**.001**	

Statistically significant values are shown in bold (*p* 0.05); *p* 0.001 indicates strong statistical significance

^**^Correlation is significant at the 0.01 level (2-tailed)

^*^p < 0.001 indicates strong statistical significance

## Discussion

Receiving a cancer diagnosis often brings profound physical and psychological challenges, leading individuals to seek coping mechanisms, including spiritual well-being. Prior research suggests that emphasizing spiritual well-being yields positive physical and psychological patient outcomes (Jafari et al., 2013). The current study provides a nuanced understanding of how the Faith sub-dimension of the FACIT-Sp interacts with fear and anxiety in the unique context of the COVID-19 pandemic for individuals living with cancer.

This study identified a significant negative correlation between Faith and fear of COVID-19 among participants. This finding underscores the critical role of Faith as a psychological resource in managing pandemic-related fears. Unlike the broader and potentially tautological conclusions about the meaning and peace sub-dimension of the FACIT-Sp, the Faith sub-dimension offers a more focused lens for exploring spiritual dimensions. This aligns with prior studies highlighting the protective role of spiritual well-being in fostering resilience and mitigating anxiety in individuals facing chronic illnesses (Gaston-Johansson et al., 2013; Heidari et al., 2019).

The current findings build on the work of researchers such as Durmus and Durar (2022), who reported a similar association between Faith and reduced anxiety in the context of COVID-19. Focusing on Faith, this study avoids the tautological implications associated with broader constructs like peace and meaning. Faith as a distinct construct highlights the active role of Faith systems and trust in a higher power as a source of strength during crises. Gashi (2020) further emphasizes the role of spiritual coping strategies, particularly Faith, in enhancing resilience during the pandemic.

Another significant observation is the association between older age and higher levels of Faith. Older adults often turn to spiritual practices and Faith serves to fulfill needs, promote growth, strengthen social connections, and find peace. This aligns with the findings of Ramezani et al. (2019) and Ghahremanian et al. (2016), who emphasized that older individuals prioritize fundamental spiritual needs such as connecting with God and finding purpose. These findings also support the observations of Durmuş and Durar (2022) regarding the stronger links between spiritual well-being and reduced anxiety among older adults. This phenomenon is likely attributed to their life experiences, well-developed coping mechanisms, and the integration of Faith into their daily lives, enhancing their resilience during crises.

This study also found that individuals hospitalized due to COVID-19 exhibited increased Faith, likely influenced by the compassionate care provided in hospital settings. Research indicates that a supportive environment characterized by respect and empathy strengthens spiritual well-being and helps patients cope more effectively with stress (Koenig et al., 2004). Providing spiritual support in healthcare settings enhances resilience and improves treatment outcomes, highlighting the need for a holistic approach to patient care.

### Limitations

While this study provides valuable insights, several limitations must be acknowledged. The cross-sectional design prevents causal inferences, and the sample may not fully represent the broader experiences of cancer patients, particularly those from diverse cultural or socioeconomic backgrounds. Additionally, the study's focus on the FACIT-Sp Faith sub-dimension does not fully capture the multifaceted nature of spirituality. Future research should explore these dynamics using longitudinal methods and include diverse populations to enhance the generalizability of the findings.

## Conclusion

This study highlights the negative relationship between the Faith sub-dimension of spiritual well-being and fear of COVID-19 among individuals with cancer. By focusing on Faith, this research provides actionable insights into spiritual dimensions that reduce anxiety and enhance resilience. These findings contribute to the growing body of literature on spirituality and mental health, emphasizing the need for integrated spiritual support in healthcare services and public health interventions.

### Research and Clinical Practice

The findings have significant implications for clinical practice, particularly in cancer care. Faith-based interventions can improve overall well-being and mental health by addressing patients' spiritual needs. Integrating spiritual counseling, creating spaces for reflection, and collaborating with spiritual care professionals are practical strategies to support cancer patients. Policymakers and healthcare practitioners may consider designing public health strategies incorporating faith-based support systems, particularly for vulnerable populations such as the elderly or individuals with chronic illnesses.

### Recommendations

Future research should include larger and more diverse populations to validate these findings and further explore how Faith contributes to resilience among cancer survivors. Healthcare providers should receive specialized training to address patients’ spiritual needs, focusing on faith-based interventions tailored to specific populations. These initiatives would foster a more comprehensive, patient-centered approach to managing chronic illnesses and related stressors, such as pandemic-related fears.
